# A unified survival-analysis approach to insect population development and survival times

**DOI:** 10.1038/s41598-021-87264-1

**Published:** 2021-04-15

**Authors:** Zhanshan (Sam) Ma

**Affiliations:** 1 Computational Biology and Medical Ecology Lab, Kunming Institute of Zoology, Chinese Academy of Sciences, Kunming, China; 2 Center for Excellence in Animal Evolution and Genetics, Chinese Academy of Sciences, Kunming, China; 3 Department of Entomology, Plant Pathology and Nematology, University of Idaho, Moscow, ID 83843 USA

**Keywords:** Computational biology and bioinformatics, Ecology

## Abstract

There are two major categories of observation data in studying time-dependent processes: one is the time-series data, and the other is the perhaps lesser-recognized but similarly prevalent *time-to-event* data (also known as *survival* or *failure time*). Examples in entomology include molting times and death times of insects, waiting times of predators before the next attack or the hiding times of preys. A particular challenge in analyzing time-to-event data is the observation *censoring*, or the incomplete observation of survival times, dealing which is a unique advantage of survival analysis statistics. Even with a perfectly designed experiment being conducted perfectly, such ‘naturally’ censoring may still be unavoidable due to the natural processes, including the premature death in the observation of insect development, the variability in instarship, or simply the continuous nature of time process and the discrete nature of sampling intervals. Here we propose to apply the classic Cox proportional hazards model for modeling both insect development and survival rates (probabilities) with a unified survival analysis approach. We demonstrated the advantages of the proposed approach with the development and survival datasets of 1800 Russian wheat aphids from their births to deaths, observed under 25 laboratory treatments of temperatures and plant growth stages.

## Introduction

Compared with its de facto standard position in biomedicine, the applications of survival analysis in entomology and ecology are *relatively* limited. In recent years, some important applications have been conducted, e.g., Drake 2006; Fox and Kendall 2006; Gienapp 2005; He and Alfaro 2000; Ma 1997; Ma and Bechinski 2008b; McLoughlin et al. 2005; Moya-Laran and Wise 2000; Tanhuanpa et al. 2001; Velema et al. 2005; Zens and Peart 2003^1–11^. Still, it seems that survival analysis can play an even broader role in entomology and ecology. This should become obvious if we simply examine the variables that survival analysis is devised to study. As a rapidly expanding subject of mathematical statistics, survival analysis studies positive random variables for describing times to events of concern, also known as survival time, failure time, or simply time-to-event. Survival analysis is therefore also known as failure time analysis or time-to-event analysis. Examples of survival time include failure times of vehicles, survival times of cancer patients, molting times of insect larvae, and certainly, the lifetimes of organisms and humans. In general, there are two major types of data from observing a time-dependent process: one type is the time-series data and the other is the time-to-event data. In entomological research, the field observations of population dynamics often collect time-series data, but in laboratory demography, life tables, bioassay, behavior biology, and some other studies, time-to-event observations such as molting, emergence, longevity, death time after chemical treatment, as well as the occurrence times of behavior or physiological events are frequently the predominant variables to observe.

Although the applications of survival analysis in entomology are expanding in recent years, it appears that the majority of these studies are ad hoc applications. We think that this state-of-the-art of survival analysis in the entomological literature is due to two factors: (*i*) Survival analysis is much more complex than many commonly used statistical approaches. This opinion was echoed by Murtaugh (2007)^12^, who criticized the often unnecessarily complex statistical analysis. However, Murtaugh (2007) also commented: "Some statistical methods are unavoidably complex, e.g., *survival analysis* and spatial statistics^12^. Presentations of such analyses will necessarily be quite technical and involved." O’Quigley (2008) commented “The subject [*survival analysis*] is very difficult and while outstanding efforts have been made across the globe in leading statistics and biostatistics departments to explain the essential ideas behind the material^13^, few would claim that students, other than the small minority already well steeled in mathematical analysis, ever really fully grasp just what is going on. This is a situation that we need be concerned about”. Although O’Quigley’s (2008) concern was focused on the measure-theoretic treatment of survival analysis and directed to mathematicians, his point should also make sense to biologists who are interested in the application of survival analysis^13^. (*ii*) It appears that few of the existing studies in literature have argued why survival analysis is particularly advantageous for entomological research based on *the unique nature of insects* (populations). We believe that proper addressing of the above two aspects should lead to even broader adoption of survival analysis in entomological research. For the first factor, it is suggested that a qualified statistician who is well-versed in survival analysis should be invited to assist in the design and data analysis of entomological experiments. For the second factor, the author first identified and elaborated in his dissertation (Ma 1997) more than two decades ago^1^. In consequent journal publications, Ma & Bechinski (2008b, 2009b, Ma 2010) reported some of the results from that dissertation (Ma 1997), including summarizing the issues related to the second factor^1,2,14,15^. Considering the necessity for understanding why survival analysis is advantageous for studying insect development and survival because of the inherent properties of insect populations, in the remainder of this introduction section, we further elaborate the key arguments reported previously (Ma & Bechinski 2008b, 2009b, Ma 1997, 2010)^1,2,14,15^. In the material and methods section, we present a more comprehensive picture of the relevant issues somewhat unique in entomology to put this study in proper context. However, due to the enormous breadth of both survival analysis (as an important branch of modern statistics) and entomology, the objective of this paper is much more focused. Specifically, we apply Cox's proportional hazards model (PHM), to study the development and survival of Russian wheat aphid (RWA), *Diuraphis noxia* (Mordvilko) under changing temperatures and plant growth stages, for both life processes, with a unified modeling approach. The Cox's (1972) PHM is well recognized as a fundamental model of survival analysis, which has been extended numerously and essentially forms a fundamental approach for survival analysis^16^. Whole monographs (*e.g.,* Therneau and Grambsch 2000, Martinussen and Scheike 2006, O’Quigley 2008) have been written to discuss this set of models and associated approaches^13,17,18^; therefore we expect that PHM and its extensions should become a major approach for modeling insect development and survival. For the general theory and statistics of survival analysis, more than a dozen monographs and textbooks, as well as numerous reviews of survival analysis have been published since the 1980s (*e.g*., Kalbfleisch and Prentice 1980, 2002; Cox and Oakes 1984; Fisher and Lin 1999; Therneau & Grambsch. 2000; Hougaard 2001; Lawless 2003; Ibrahim et al. 2005; Martinussen and Scheike 2006; Aalen 2008; O’Quigley 2008; Ma & Krings 2008a, 2008b; Ma et al. 2008; Kleinbaum 2012; Keiding 2014; Amico & Keilegom 2018; Grigorios et al. 2019; Shrestha et al*.* 2019)^13,17–34^.

Although we recognize that, in recent years, there are alternative probabilistic (statistical) approaches to dealing with the censoring in insect development and survival times (most notably by Régnière et al. 2012; Rukke et al. 2018; Grant et al. 2020)^35–37^, we believe that survival analysis approaches are more straightforward and relatively easy to apply, in particularly with extensive supports from open source R software (https://cran.r-project.org/web/views/Survival.html; https://rviews.rstudio.com/2017/09/25/survival-analysis-with-r/).

## Material and methods

### Experimental data of Russian wheat aphid (RWA) development and survival

The RWA development and survival data are from our laboratory experiments which involve the observations of 1800 RWA individuals in a factorial arrangement of five temperature and five barley plant-growth stage regimes with a total of 25 treatments. Each treatment has 72 RWA individuals as replicates. The experiment was designed to investigate the influence of temperature and barley plant-growth stage treatments on RWA development, survival and reproduction in controlled environment growth chambers. Temperature treatments were 8–1 °C, 17–10 °C, 23–16 °C, 28–21 °C, and 33–26 °C, fluctuating on a 14:10-h rectangular-wave cycle. The photoperiod was 14 *vs*. 10 (light *vs*. dark) for all treatments, with the higher constant temperature during the light phase and the lower temperature during the dark phase. Mean temperatures weighted by photoperiod were 5.1 °C, 14.1 °C, 20.1 °C, 25.1 °C and 30.1 °C, respectively. Barley plant-growth stages were two-leaf, tillering, flag leaf, inflorescence and soft dough, respectively corresponding to 12, 23, 39, 59, and 85 on the Zadoks (1974) scale^38^. More detailed information on the experiment design can also be found in Ma (1997) and Ma & Bechinski (2008a, 2008b)^1,2,39^.

For analysis, we divided the life cycle of the RWA into 9 stages: first to fifth instar (abbreviated as *1st-5th*), pre-reproductive period (from the time of last molting until the first nymphal production, designated *Pre_R*), immature period (*1st* + *2nd* + *3rd* + *4th* + *5th,* designated *immature*), mature (*immature* + *R_age*, designated *mature*), adult (from the time of last molting until death, designated *adult*). We also treat lifespan as a special variable, i.e., time from birth to death, designated *lifespan*. There is a response time *T* associated with each RWA stage and the *lifespan*; *T* is either development time (for individuals that successfully developed from one stage to the next), or death time (for individuals that died within the stage), depending on the state indication variable (short as 'state variable' or 'state'). The unit for time (*T*) is calendar day (24 h). For stages other than *adult* and *lifespan*, if the state indication variable takes a value 1, then *T* is developmental time; if state is 0, then *T* is the death time or other censored time (e.g. lost accidentally in observation). In contrast, for the *adult* and *lifespan* stages, if state is 1, then *T* is death time of an individual; if state is 0, then *T* records the time when observation stopped due to some laboratory handling accident before the individual *naturally* died. Further information on the laboratory experiments is also described in Ma (1997), and Ma & Bechinski (2008a, 2008b)^1,2,39^.

### Unified survival analysis approach to insect development and survival

#### Issues of censoring in entomological research

Censoring occurs when the failure times of some individuals within the observation sample cannot be observed. Censoring is often unavoidable in time-to-event studies. A patient in a clinical trial may be withdrawn from the study after a period of participation; similarly, insects under observation may be lost tracks due to accidental events such as operational faults. Such kind of censoring belongs to the so-termed *random censoring*. In other cases, observing all individuals for the full time course to failure (such as the occurrence of death) is too costly or unacceptable, leading to the so-terms *right censoring.* In other situations, the process may have been going on but unnoticed prior to formal study, and consequently a starting point has to be selected, such as the exposure to some newly discovered risks, or the occurrence of a new infectious disease. This last category of censoring is known as *left censoring*.

All three censorings exemplified above may occur in entomological experiments. Whereas the censorings discussed so far might be avoided or minimized, we realize that, in the study of insect populations, even with a perfect experiment design being perfectly executed, at least two types of *natural* censoring mechanisms seem uncontrollable thanks to the very nature of insect development and instarship. Two examples are presented here: (*i*) In a life table study, when a cohort of insects is observed, the insect development (molting, emergence, etc.) or survival (death) are typical examples of time-to-event or failure time random variable. This is not the focal point of our arguments. The point is that some insect individuals may die and never emerge from the observed instar or stage. From the perspective of observing insect development, the data may be censored due to the “*premature*” death events. How long it would have taken for those prematurely dead individuals to complete their developments is hardly knowable. (*ii*) It is well known that the number of instars in an insect species may be different among individuals of the same population; one may never know the exact number of instars an individual can potentially experience if it dies prior to reaching adult stage. For example, in the case of RWA, the majority of individuals has 4 instars, but 2, 3, 5 are also possible. If a RWA nymph died before reaching the adult stage, we may never know how many instars this prematurely dead individual would pass through. Hence, unless zero mortality in immature stages is possible, censoring in studies of insect developments occurs *naturally* and is incontrollable. Therefore, insect development and survival are *dependent* in the sense that in order to develop to the next stage, an insect individual must survive through the current stage. Such kind of dependence can be addressed ideally with conditional probability models in survival analysis including Cox proportional hazards models, which is applied to modeling the development and survival of RWA in this paper.

With most statistical methods other than survival analysis, censoring presents a dilemma. If a researcher chooses to exclude the censored observations, the resulting sample may be too small to conduct proper statistical analysis. Even if the resulting sample is large enough, the parameters estimated are biased, and there is no well-established statistical procedure to quantify the bias, because there is no guarantee that the censored individuals can be represented by the remaining sample. On the other hand, if the censored individuals are included, although compartmental modeling (using a probabilistic approach, or invoking differential equations) might be useful so that the numbers of individuals in the different instars (compartments) are modeled over time with estimation of the rates of transition, there are no objective procedures to process their "partial" lifetime information, which again may introduce bias without even knowing the degree of bias introduced.

How significant can the difference be between the two schemes—one with censored individuals excluded before applying survival analysis, and the other processed with survival analysis directly (*i.e.*, the partial information of the censored individual is preserved)? Ma (1997, 2010)^1,15^ and Ma & Bechinski (2008b, 2009b)^2,14^ treated the prematurely dead RWA individuals as censored with survival analysis approach, and found that the difference between two treatments: survival analysis *vs*. excluding the premature dead individuals range from 4%–25% in the estimate of median development, depending on the *severity of censoring* (death rates)^15^. The treatment resolves the previously identified dilemma because survival analysis has developed effective procedures and methods (based on the asymptotical theory or more recently on the counting stochastic process) that can properly extract the partial information in those censored data.

While the previous type of censoring due to premature death or variable instarships seems more likely to occur in insect demography and phenology studies under laboratory conditions, there is another subtle but hardly avoidable censoring mechanism, which is termed as *interval censoring* in survival analysis and it may be more likely to be encountered in field insect research such as life table study by sampling insect population periodically. This type of censoring is required because sampling is discrete and linear, whereas the process under investigation is continuous and possibly nonlinear with respect to time, which makes the precise recording of the survival times for *all* individuals in an experiment impossible. With interval censored data, each event time is then only known to lie in some interval, and the precise time to an event is often not known due to the limited sampling points.

Ma & Bechinski (2008b, 2009b, Ma 1997, 2010) argued that^1,2,14,15^, whenever time-to-event data is in concern, survival analysis can be harnessed to perform the two fundamental statistical analyses in lieu of traditional statistical methods, that is: (*i*) *hypothesis testing*—replacing procedures such as significance testing, ANOVA, life tables analysis etc.; (2) model-parameter estimation—replacing conventional regression modeling. The prevalence of time-to-event data as one of the two major categories of time-dependent process data (the other is time-series data as noted previously), as well as the fact that survival analysis is developed to study time-to-event variables with observation censoring, make a very strong case for entomologists to adopt survival analysis as an appropriate statistical tool. In a previous study, we demonstrated the use of survival analysis for *hypothesis testing and life table analysis* (Ma 2010)^15^. In the present paper, we demonstrate the second area—*model*-*parameter estimation*. Specifically, we try to show that survival analysis offers a unified approach to model both insect *development* and *survival*.

#### Survival Analysis and Proportional Hazards Model (PHM)

##### Survivor, hazards and probability density functions

Given response time (survival or failure time) *T* of a subject, three functions are usually used to describe the random variable (*T*): the survivor function, the probability density function, and the hazard function.

The *survivor function S*(*t*) is defined as the probability that *T* is at least as great as a value *t*; that is,

1 $$S(t) = P(T \ge t)\quad t > 0.$$

The survivor function is actually *1*′s complement of the distribution function of random variable (*T*), that is, *S*(*t*) = 1–*F*(*t*), where *F*(*t*) is the distribution function of *T*.

The *probability density function* (*p.d.f*) of *T* is

2 $$f(t) = \mathop {\lim }\limits_{{\Delta t \to 0^{ + } }} \frac{{P(t \le T \le t + \Delta t)}}{{\Delta t}} = - \frac{{dS(t)}}{{dt}}.$$

Conversely,$S(t) = \int_{t}^{\infty } {f(u)du}$ and *f*(*t*) ≥ 0 with $\int_{0}^{\infty } {f(t)dt = 1.}$

The *hazard function* specifies the *instantaneous* rate of failure at *T* = *t*, conditional upon survival to time *t*. It is defined as

3 $$\lambda (t) = \mathop {\lim }\limits_{{\Delta t \to 0^{ + } }} \frac{{P(t \le T < t + \Delta t|T \ge t)}}{{\Delta t}} = \frac{{f(t)}}{{S(t)}}.$$

The relationships among *S*(*t*), *f*(*t*), and λ(*t*) are expressed as follows:

4 $$\lambda (t) = \frac{ - d\log S(t)}{{dt}}$$

5 $$S(t) = \exp \left( { - \int_{0}^{t} {\lambda (u)du} } \right)$$

6 $$f(t) = \lambda (t)\exp \left( { - \int_{0}^{t} {\lambda (u)du} } \right).$$

##### Proportional hazards model (PHM)

Cox (1972, 1975) proposed the proportional hazards model (PHM)^16,40^

7 $$\lambda (t,\,z) = \lambda_{0} (t)\exp (z\beta ) = \lambda_{0} (t)\exp (\beta_{1} z_{1} + \beta_{2} z_{2} + ...\beta_{n} z_{n} ),$$

where λ(*t*, *z*) denotes the *hazard function* at time *t* for an individual with the characteristic represented by the covariate vector *z* of *n* elements. In entomological research, examples of *z* may include environmental factors (temperature and plant growth stage in this paper) that influence the development and survival of insects. Here λ_0_(*t*) is an arbitrary unspecified baseline hazard function for continuous time *t*. The hazards function λ(*t*, *z*) is a product of an underlying age-dependent risk, λ_0_(*t*) (baseline hazard function) and another factor, exp(*zβ*), which depends on covariates *z* and the vector *β* of parameters. Baseline hazard function λ_0_(*t*) is the hazard function for individuals on which covariates have “neutral effect”—the values of covariates are equal to either zero or to their averages (an example is shown later) depending on the model form adopted. The PHM estimates the risks of other groups in relation to this baseline. Other specifications of the hazard relationship are possible (*e.g*., λ(*t*, *z*) = λ_0_(*t*) + *zβ*), but the problem with these alternatives is the mathematical possibility of predicting negative hazard rates, which then requires extra constraints on estimation procedures to ensure positive values.

The PHM invokes two assumptions. The first is the *proportionality assumption,* that there is a multiplicative relationship between the underlying hazard function and the log-linear function of the covariates such that the ratio of hazard functions for two individuals with different sets of covariates is constant in time (from which the PHM derived its name). The second assumption is that effects of covariates on the hazard function are log-linear.

The conditional (with respect to the covariate vector *z*) probability density function of *T* given *z* for the PHM is

8 $$f(t;\,z) = \lambda _{0} (t)\exp (z\beta )\exp \left[ { - \exp (z\beta )\int_{0}^{t} {\mathop \lambda \nolimits_{0} (u)du} } \right].$$

where λ_0_(t) is the baseline hazard function as explained previously, *z* is the vector of covariates (*e.g*.. air temperature and crop growth state in this study), and *β* is the vector of Cox’s PHM regression coefficients (parameters).

The conditional survivor function (or simply called the survivor function) of *T* given *z* for the PHM is

9 $$S(t;\,z) = [S_{0} (t)]^{\exp (z\beta )} ,$$

where

10 $$S_{0} (t) = \exp \left[ { - \int_{0}^{t} {\lambda _{0} (u)du} } \right].$$

*S*_0_(*t*) is called the *baseline* survivor function; it is computed for the default categories of the covariates (*e.g*., average temperature and plant growth stage in the case of this study). Therefore, the survivor function of *t* for a covariate vector *z* is obtained by raising the baseline survivor function *S*_0_(*t*) to a power. The usefulness of Eq. (9) is that one can predict survivor probabilities under different covariate values.

If λ_0_(*t*) is arbitrary, this model is sufficiently flexible for many applications. There are two important generalizations that do not substantially complicate the estimation of *β*, but broadly expanding their applications: the stratified proportional hazards model and the proportional hazards model with time-dependent covariates.

In the stratified version, the function λ_0_(*t*) is allowed to vary in specific subsets of the data. In particular, the population is divided into *r* strata wherein the hazard *λ*_*j*_(*t*; *z*) in the *j-*th stratum depends on an arbitrary shape function *λ*_*0j*_(*t*). The model can be written as

11 $$\lambda_{j} (t,\,z) = \lambda_{0j} (t)\exp (z\beta )\quad j = {1},{ 2}, \ldots ,r.$$

This generalization is useful when the covariates do not seem to have a multiplicative effect on the hazard function. Here the range of those variables can be divided into strata where only the remaining regression variables contribute to the exponential factor in Eq. (11).

The second generalization to the PHM is to allow the variable *z* to depend on time itself, without (Eq. 12) or with (Eq. 13) stratification:

12 $$\lambda [t,\,z(t)] = \lambda_{0} (t)\exp [z(t)\beta ],$$

13 $$\lambda_{j} [t,\,z(t)] = \lambda_{0j} (t)\exp [z(t)\beta ]\quad j = 1,2, \ldots ,r.$$

The estimation of *β* depends only on the rank ordering of the variable vector *z* and is invariant with respect to the monotonic transformation on the dependent variable, *i.e*., survival time. The procedure used to estimate *β* is to maximize the so-called *partial likelihood functions* as described by Cox (1975), Kalbfleisch & Prentice (1980, 2002) and Kleinbaum and Klein (2012)^19,20,30,40^. BMDP™ 2L program, Survival Analysis with Covariates (BMDP 1993)^41^, was used to construct the proportional hazards models. The input data set was as described in Ma (1997)^1^. Finally, as an example, we use 1989 air temperature and barley plant-growth stage data from Moscow, ID., reported by Elberson (1992)^42^, as inputs to run the proportional hazards model for survival of RWA during the entire life cycle (i.e., model for *LifeSpan* stage). We further used coxphf function in the Survival package of open source R-Project to cross-verify the results from BMDP software. The information of Survival package, which is the cornerstone of R implementation of survival analysis, can be accessed at: (https://cran.r-project.org/web/views/Survival.html).

## Results and discussion

### Cox PHM with temperature and barley plant-growth stage as covariates

Table 1 shows results of fitting Cox PHM (proportional hazard model) for 1^st^ instar RWA (Russian wheat aphid) nymph using temperature and plant growth stage as covariates.

**Table 1:** Results of fitting Cox’s proportional hazards model (PHM) for the development of the *first* instar RWA nymph with temperature and plant-growth stage as covariates.

Variable (covariate)	Coefficient (β)	Standard error
Parameter (β) of Cox’s proportional hazards model*		
Temperature	0.0815	0.0045
Stage	− 0.0029	0.0012

Development (molting) time (days)	Baseline survival	Baseline cumulative hazard
**Time Variable is the 1st-Instar RWA nymph**		
1	0.9982	0.0018
2	0.9622	0.0385
3	0.7497	0.2880
4	0.5524	0.5936
5	0.4292	0.8457
6	0.3540	1.0385
7	0.3065	1.1824
8	0.2616	1.3408
9	0.2132	1.5454
10	0.1347	2.0047
11	0.0735	2.6106
12	0.0518	2.9612
13	0.0295	3.5248
14	0.0181	4.0113
15	0.0107	4.5413
16	0.0077	4.8688
17	0.0065	5.0315
18	0.0048	5.3378
19	0.0024	6.0522
20	0.0015	6.4954
21	0.0012	6.7522
22	0.0006	7.3403
23	0.0004	7.7417
24	0.0002	8.5588
28	0.0001	9.0428
30	0.0001	9.6274
31	0.0000	10.1221

Number of data cases = 1800.

Log likelihood = – 6939.7053.

Global Chi-square = 364.73.

Degree of freedom = 2.

*P*-value < 0.00001.

*Risk type is Log-linear.

In Table 1, the “NUMBER OF CASES READ = 1800” is the number of RWA individuals observed in the experiment. The header “RISK TYPE IS LOGLIN” indicates that the *risk* function is log-linear which is the default for Cox PHM as described by Eq. (7), other available risk types include LINEAR, COMBINATION and USER defined. When the log-linear risk type is chosen, the covariate means are subtracted from observation values. Therefore, the values of the baseline survivor function are the survivor function when the covariates values take their means. In our study, the means of covariates temperature and plant growth stage are 18.88 °C and 43.6 (Zadoks scale) respectively.

The logarithm of the maximized partial likelihood function and the global chi-square statistic (and its degree of freedom and *p*-value also) are listed. The global chi-square statistic tests the null hypothesis that all regression coefficients equal zero. Listed next for each covariate is the computed parameter estimates (COEFFICIENT), and their asymptotic standard errors. The regression coefficients indicate the relative effect of the covariate on the hazard function. A positive coefficient increases the value of the hazard function and therefore, indicates negative effect on response time. A negative one should be interpreted conversely. For the development of 1*st* instar RWA, the positive coefficient corresponding to temperature indicates that the increasing temperature will reduce the response times—the development times. In other words, temperature tends to accelerate the development of the1*st* instar RWA. Conversely, the plant-growth stage decelerates the development of the 1*st* instar RWA, because coefficient corresponding to plant stage is negative.

The middle section of Table 1 lists the values of the baseline survivor and cumulative hazard functions. The conversion factor is in the expression exp(β′z), which is the exponent used to convert the estimated baseline survivor function *S*_0_(t) into the survivor function described by (9), given a covariate vector z. For example, given covariate values 20.1 °C and 12 for temperature and crop stage, respectively, then the conversion factor is calculated as exp{[0.0815*(20.1–18.88)–0.0029*(12–43.6)]} = 1.207, where 18.88 and 43.6 respectively are mean temperatures and crop stages from our lab experiment. With the conversion factor for the particular covariates values and the baseline survivor function values, one can predict the survivor function value of RWA at any time. For example, under 20.1 °C and at Zadoks plant-growth stage12, the survivor function value on day 10 is *S*[10, (20.1, 12)] = *S*_0_(10)^1.207^ = 0.1347^1.207^ = 0.09 = 9%. That value means that after 10 days, there is a 91% (100–9%) probability that a newly born RWA nymph will have molted (*responded*) and developed to the next instar at 20.1 °C and on a Zadoks’ scale 12 (two-leaf) barley plant.

**Figure 1:**
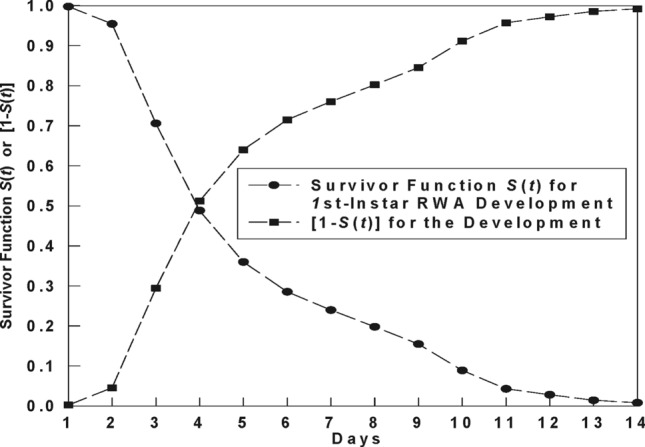
Survivor function [*S*(*t*)] and cumulative development probability [1 − *S*(*t*)] modeled with Cox PHM for the development of the *first*-instar RWA nymph.

To further illustrate the overall picture of the modeling RWA development with PHM, Fig. 1 is drawn from the fitted PHM model for the development of *1st*-instar RWA. Let us first represent the Table 1 as a mathematical model, which contains all the information necessary for representing the PHM model for the development of the *1*st-instar RWA. The baseline survivor function values are from Table 1 (column 3) and the two parameters of PHM are [β_1_, β_2_] = [0.0815, − 0.0029], corresponding to temperature and plant stage, respectively, which are also taken from Table 1 and also listed in Table 2. By substituting for parameters [β_1_, β_2_] with [0.0815, − 0.0029] in Eq. (9), we obtain the following model for the development of the *1*st-instar RWA:

14 $$S(t|Temperature,PlantStage) = S_{0} (t)^{\exp [0.0815*(Temperature - 18.88) - 0.0029*(PlantStage - 43.6)]} ],$$

where *S*_0_(*t*) is the baseline survivor function (column 3 in Table 1).

**Table 2:** Proportional hazards models (PHM) for RWA development and survival.

RWA stage	PHM for	*β*_1_ (Temperature) Coeff. (Std. Err.)	*β*_2_ (Crop Stage) Coeff. (Std. Err.)	Chi-Square	*P* value
1st	Development	0.0815 (0.0045)	− 0.0029 (0.0012)	364.70	0.0000
	Survival	0.1510 (0.0066)	0.0105 (0.0014)	657.53	0.0000
2nd	Development	0.0990 (0.0052)	− 0.0031 (0.0014)	417.25	0.0000
	Survival	0.1440 (0.0118)	0.0082 (0.0029)	158.71	0.0000
3rd	Development	0.0914 (0.0055)	− 0.0014 (0.0014)	309.03	0.0000
	Survival	0.1735 (0.0176)	0.0089 (0.0042)	120.71	0.0000
4th	Development	0.1336 (0.0079)	− 0.0032 (0.0019)	312.06	0.0000
	Survival	0.1932 (0.0246)	0.0143 (0.0050)	68.52	0.0000
5th	Development	0.0693 (0.0215)	− 0.0039 (0.0062)	13.30	0.0013
	Survival	0.0542 (0.0295)	0.0024 (0.0082)	3.45	0.1783
Pre_R	Development	0.0255 (0.0050)	− 0.0030 (0.0015)	36.12	0.0000
	Survival	0.6283 (0.0724)	0.0040 (0.0021)	543.27	0.0000
Immature	Development	0.1551 (0.0044)	0.0055 (0.0009)	1372.84	0.0000
	Survival	N/A*	N/A	N/A	N/A
Mature	Development	0.2321 (0.0080)	− 0.0023 (0.0015)	1001.85	0.0000
	Survival	0.1216 (0.0051)	0.0099 (0.0012)	645.37	0.0000
Adult	Development	N/A	N/A	N/A	N/A
	Survival	0.1285 (0.0067)	0.0038 (0.0014)	396.08	0.0000
LifeSpan	Development	N/A	N/A	N/A	N/A
	Survival	0.1358 (0.0044)	0.0087 (0.0009)	1063.88	0.0000

N/A refers to stages where there is only one event (development or survival) that makes sense biologically, or both development and survival refer to the same event. For example, the completion of development of the adult stage is equivalent to the death of the adult. *β*_1_ = (*β*_1,_ *β*_2_) are the regression coefficients of Cox’s PHM, as defined in Eqs. (8–11).

The reason for subtracting the 18.88 and 43.6, which are the *mean* temperature and plant growth stage used in modeling, respectively, in Eq. (14) has to do with the standardization of the covariates such that when covariates take their *means* (18.88 and 43.6 in this case) , the survivor function equals its baseline value *S*_0_(*t*). This also is related to the choice of risk type (Log-linear here) as explained previously in the context of Table 1.

Figure 1 is then drawn with Eq. (14); it contains two graphs, one is drawn from the above model for *S*(*t*), and the other is simply the [1 − *S*(t)]. The temperature and plant growth stage used to draw the graph are 20.1 °C and at Zadoks-Scale of 12 (two-leaf stage), respectively. As explained previously, survivor function *S*(*t*), when built for RWA development, it implies the probability that a RWA individual has not yet finished the development at time *t* ("*still surviving the development event*"), conditional on the covariates Z = [*Temperature*, *PlantStage*]. For a RWA *population*, this probability implies that there are *S*(*t*) percentage of RWA that have not yet finished the development of the *1*st-instar at time *t*. Therefore, [1 − *S*(t)], which might be more intuitive for measuring insect development, is the (cumulative) probability that an individual has finished development of the stage at time (*t*), or the *cumulative percentage* of individuals in a population that have finished the development.

Table 2 lists the parameters of proportional hazards models for both development and survival of all ten RWA stages. The baseline survivor and hazard functions are not listed to save space. Note that both the *development* time and *survival* time of a stage were measured from the beginning of that stage. The β_1_ & β_2_ are the regression coefficients for temperature and crop stage respectively. The values within parentheses are the standard errors of the coefficients. The extremely high global Chi-square and low associated *p*-value in Table 2 demonstrate that the models fit to the data of all stages exceptionally well, except for the survival of 5-th instar nymphs. Only the PHM for survival of 5-th instar has a higher *p* value of 0.18. Because few RWA individuals developed through a 5th nymphal instar (most instead developed through four nymphal instars before reproducing), the sample size is very small and the resulting *p*-value is not reliable. We believe it unlikely that the survival pattern of 5-th instars would differ from those other RWA nymphal instars. Therefore, we conclude that all estimated parameters (or regression coefficients) of the proportional hazards models for RWA are significantly differ from zero, and that the corresponding PHMs are reliable except for survival of the 5-th instar. The positive values of β_1_ for development indicate that increasing temperature increases the ‘hazard’ of the development event and so reduces development time. Conversely, the negative values of β_2_ for development imply that increasingly older plant growth stages delay the development event. Similarly, because both β_1_ and β_2_ parameters from the proportional hazards models for RWA stage survival are positive, this means that increasing either temperature or plant-growth stage increases the “hazards” of survival, and so are unfavorable to RWA survival.

### Stratified PHM (proportional hazards models)

The results in previous section indicate that the basic PHM is sufficient to model RWA development and survival. In this section, to demonstrate the extended PHMs, we also build stratified PHMs [Eq. (11)]. Table 3 lists the results of fitting proportional hazards models stratified by either temperature or plant-growth stage as a covariate. The advantage of these stratified models is that the covariates used for data stratification need not affect hazards multiplicatively. However, there is a disadvantage too: for each stratum, there is a set of baseline hazard and survivor functions, and these make computation of survivor function values more tedious. Note that the model parameters indicate the same trends in covariate effects on development and survival as those results from non-stratified proportional hazards models. This suggests that for the two-covariate factors (temperature and plant growth stage) we studied in this paper, stratification is optional. Nevertheless, for general study of insect population dynamics, when more covariate factors are considered and some of them may not act multiplicatively, then stratified PHMs should be adopted.

**Table 3:** Results of fitting stratified proportional hazards models for RWA development and survival.

Models		Stratified by temperature			Stratified by crop stage		
RWA stage	Models for	β (stage)	Chi-Square	*p* value	β (temperature)	Chi-Square	*p* value
1st	Development	− 0.0042	12.34	0.0004	0.0815	351.61	0.0000
	Survival	0.0104	54.37	0.0000	0.1510	611.77	0.0000
2nd	Development	− 0.0061	19.17	0.0000	0.0968	353.39	0.0000
	Survival	0.0089	8.63	0.0033	0.1468	161.00	0.0000
3rd	Development	− 0.0055	13.91	0.0002	0.0909	264.12	0.0000
	Survival	0.0073	2.77	0.0960	0.1851	125.33	0.0000
4th	Development	− 0.0055	8.39	0.0038	0.1300	270.07	0.0000
	Survival	0.0156	8.68	0.0032	0.2020	71.13	0.0000
5th	Development	− 0.0151	5.17	0.0230	0.0618	6.40	0.0114
	Survival	0.0066	0.41	0.5209	0.0719	4.96	0.0260
Pre_R	Development	− 0.0029	3.84	0.0500	0.0282	28.71	0.0000
	Survival	0.0035	2.84	0.0920	0.6257	502.07	0.0000
Immature	Development	0.0037	16.05	0.0001	0.1522	1327.10	0.0000
	Survival	N/A*			N/A		
Mature	Development	− 0.0080	27.39	0.0000	0.2337	858.09	0.0000
	Survival	0.0107	83.91	0.0000	0.1212	622.41	0.0000
Adult	Development	N/A	N/A	N/A	N/A	N/A	N/A
	Survival	0.0074	26.10	0.0000	0.1329	390.33	0.0000
LifeSpan	Development	N/A	N/A	N/A	N/A	N/A	N/A
	Survival	0.0098	108.47	0.0000	0.1973	921.29	0.0000

N/A refers to stages where there is only one event (development or survival) that makes sense biologically, or both development and survival refer to the same event. For example, the completion of development of the adult stage is equivalent to the death of the adult.

### An illustrative example of proportional hazards model

The proportional hazards models quantitatively describe relationships between survivor functions (hazards functions) with RWA age and environmental factors, with respect to the development and survival of RWA. In practice, these models can be used to predict RWA *age-specific survival* and *development* rates under variable environment conditions. Here, we illustrate how the proportional hazards model can be applied to estimate RWA survivorship during the life cycle (last column, *LifeSpan*, in Table 2).

The proportional hazards model for RWA lifetime survival is then obtained from Eq. (9) with the corresponding parameters for *LifeSpan* stage in Table 2 as following:

15 $$S\left( {t, \, temp, \, stage} \right) \, = S_{0} (t)^{\exp [0.1358*(temp - 18.88) + 0.0087*(stage - 43.6)]}$$

where *t, temp and stage* are RWA age in days, temperature and barley plant-growth stage, respectively. *S*_0_(*t*) is the baseline survival function. *S*(.) is the age-specific, temperature and plant-stage dependent survivor function for RWA lifetime survival.

**Figure 2:**
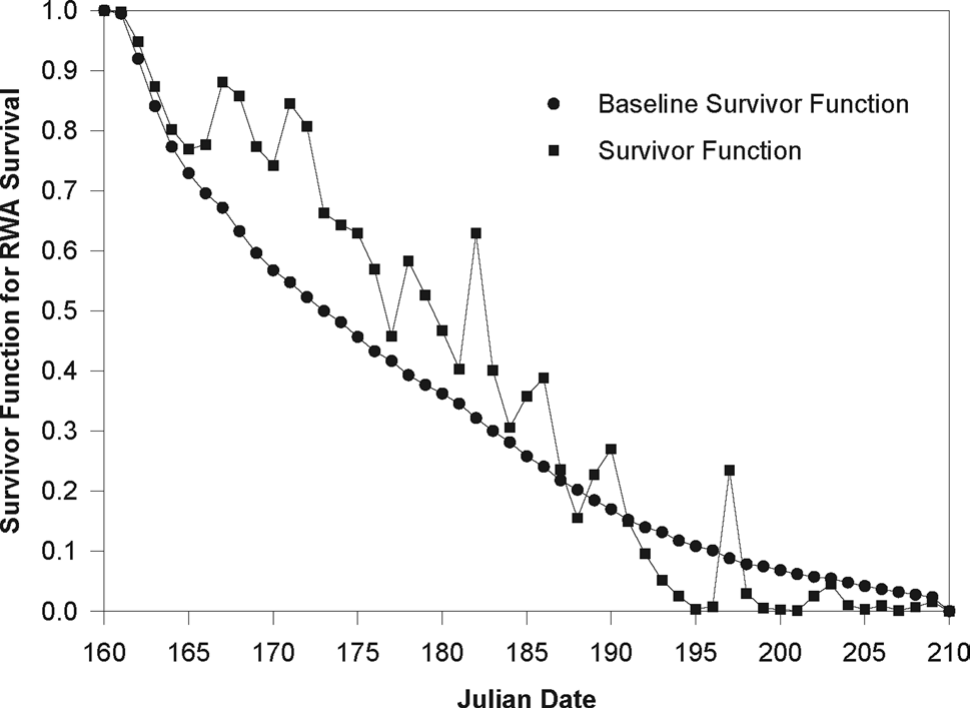
Predicted Cox conditional survivor function values for the RWA lifetime (survival time).

In practice, we are more interested in the real-time or calendar time prediction of insect development and survival rates (probabilities with survival analysis). The average air temperature and plant growth stage for each calendar date can be obtained from meteorological and phenological observations. If forecasting data for air temperature and plant growth stage are available, then survival analysis models such as PHM models built in this paper can be used to predict daily Cox conditional development and survival probabilities of insects. Figure 2 is such a forecasting curve drawn for the lifetime survival of RWA (Eq. 15). The x-axis is the Julian date from 160 to 210, and y-axis show both the baseline survival function (smooth one) and the *real* survival; the latter fluctuates due to the effects of variable temperature and plant growth stage. The air temperature data is from Elberson (1992)^42^, which documented the air temperature in Moscow, ID, USA in 1989 from Julian date 160 to 210. The raw plant growth stage data in the same Julian dates are also from Elberson (1992)^42^, from which Ma (1997) built the following logistic equation: ZGS = 92.73/[1 + exp(16.24–0.09*Julian-Day)], where ZGS is the Zadoks et al.(1974) growth stage scale^1,38^. With these temperature and barley plant-growth stage data inputs, the calculated survivor function values from Eq. (15) are plotted against Julian dates in Fig. 2.

Figure 2 shows both baseline and actual survivor functions. The effects of time-varying temperature and plant growth stage associated with each Julian date accounts for the fluctuation of actual survivor function values. The practical usefulness of the age-specific and environment-dependent survivor function models such as this one adopted here is clear in the context of a simulation model for RWA population dynamics as constructed in (Ma & Bechinski 2008b)^2^.

## Discussion

In previous sections, we try to demonstrate, with one of the most important models in survival analysis—Cox’s proportional hazards model (PHM), that survival analysis can be a very effective modeling tool for describing insect development and survival with the same method. A fair question is, given there is already a large set of models for insect development and mortality modeling, why does one bother to adopt the PHM or what are the potential benefits for this new approach? We expect there are, at least, following four potential advantages from using survival analysis:

First, censoring poses a dilemma to traditional statistical methods. Particularly, in modeling insect development, traditional modeling approaches do not have a well-justified approach to deal with those individuals who died before completing the development (of an instar or stage). Survival analysis such as the PHM has built-in parameter estimation procedures (*e.g*., partial likelihood estimation) to “extract” the partial information carried by those individuals who prematurely died. In other words, when modeling insect development, the premature death events, are treated as censoring. In our opinion, this is the most natural mathematical treatment we are aware of for dealing with this kind of "natural censoring" due to unavoidable premature-death events.

Second, survival analysis such as the PHM presents probability (for development or survival), rather than the simple rate of survival or development. This has two very important ramifications: (*i*) the probability expressed in *survivor function* (sensu statistics), which is different from the term "survivor" (sensu biology) associated with mortality or death events, is mathematically much more rigorous and conducive for modeling and analysis than the simple development/mortality rate. (*ii*), *survivor function* can be explained at both individual and population levels. At individual level, it is the probability of an insect to emerge from an instar or stage (complete development). With the PHM, what we obtain is actually *conditional probability* that depends on environmental covariates (such as temperature, plant growth stages). At population level, *survivor function* can be explained as the proportion of a population that can finish the development. It is clear that with this approach, individual variability, a fundamental property of insect development, and the collective (or emergent) property at the population level are unified. Apparently, advantages for modeling insect survival (mortality) with survival analysis can be argued similarly as we just did for modeling development.

Third, unlike traditional insect phenology models, which usually only considers temperature, it is extremely convenient to incorporate other environmental factors such as plant stage, humidity in the modeling of insect phenology and survival.

Fourth, survival analysis such as the PHM for insect development and survival (mortality) can be conveniently used to build a simulation model for population dynamics. This is because survivor functions for insect development and survival offer flexible and powerful conditional probability models contingent upon environment covariates. The only missing component from survival analysis is the reproduction model, which can be built with approaches such as the same-shape distribution modeling (Ma and Bechinski 2009a, 2009b)^14,43^. A simulation model based on Cox PHM modeling has been reported by Ma and Bechinski (2008b)^2^.

The flexibility and power of Cox PHM have naturally led to extensive studies on the residual diagnostics of the model. In this study, we adopted three metrics: *p* value, Chi-square test, and standard error of parameters to determine the goodness of the modeling fit. These metrics show that the fitted models explain the RWA datasets well. However, it should be noted that there are more powerful and special residual diagnostic techniques available to test the suitability of Cox PHM to data. These residual diagnostic techniques (metrics) include Cox-Snell residuals, Deviance residuals, Martingale residuals, and Schoenfeld residuals. Although they are termed residual diagnostics, their computations are much more complex and different from what traditional residual analysis does because in Cox PHM models there is no obvious counterpart of the difference between actual and predicted values of the dependent variables, which is the foundation of traditional residual analysis. Accordingly, their utilizations may also be different. For example, Martingale residual analysis cannot be utilized to determine the goodness of model fit; instead, it can be a very useful tool for detecting outliers in data or for determining the function form of covariates. Therefore, residual analysis for Cox PHM implicates more advanced survival analysis topics. Fortunately, more recent statistical software packages have implemented these residual diagnostic tools, but users still must understand their statistical meanings first.

In perspective, the latest advances of survival analysis are focused on multivariate survival analysis and Bayesian survival analysis (e.g., Hougaard, 2001; Ibrahim et al. 2005; Aalen 2008; Duchateau & Janssen 2008; Ma & Krings 2008a, 2008b; Ma et al. 2008; O’Quigley 2008)^13,23,25–29,44^. They should have even greater potential in entomology. For example, it is well established that the insect oviposition period, which is the effective reproduction lifetime, is more important to achieve the potential maximum reproduction capacity than the adult lifespan itself, and the two random variables (oviposition period and adult lifespan) may be causally linked. Therefore, multivariate survival analysis, which has advanced to study multiple lifetime variables as well as their dependence, can be applied to study the reproduction period and adult lifespan as well as the influences of the environmental factors on both of them. With the inclusion of reproduction, we obtain an integrated multivariate survival analysis modeling framework that considers survival, development and reproduction simultaneously, which would be an extension to the methodology used in the current paper, where we can only integrate survival and development. Besides dependence modeling, multivariate survival analysis has developed another powerful modeling methodology—frailty modeling—that may possess the potential to revolutionize the data analysis and modeling in insect population ecology. Actually, the mathematical study of frailty originated in the study of aging and demography of humans, and its initial objective was to demonstrate that the population hazard rate (measurement of mortality risk) can be significantly different from the hazard rates of the individuals from the population due to the effects of the frailty, which is the individual heterogeneity that are not observed or not observable in the study (Duchateau & Janssen 2008, Ma & Krings 2008a, 2008b, Ma et al. 2008)^27–29,44^. Obviously, frailty (individual heterogeneity) abounds in insect populations because the individual variation in development, survival and reproduction, as well as their response or tolerance to environment is a fundamental biological reality. For instance, one major objective of the same-shape distribution modeling for insect reproduction is to account for the individual variation in reproduction capacity (Ma and Bechinski 2009a)^43^, and frailty modeling may offer an alternative approach to the same-shape distribution methodology. One benefit from frailty modeling is that, as mentioned previously, we can model the three major modules of population dynamics: development, survival and reproduction, under a unified modeling methodology by taking advantages of the dependence and frailty modeling in multivariate survival analysis.
